# Risk Factors for Incident Dementia Among Older Cubans

**DOI:** 10.3389/fpubh.2020.00481

**Published:** 2020-09-10

**Authors:** Geeske Peeters, Arianna Almirall Sanchez, Jorge Llibre Guerra, Brian Lawlor, Rose Anne Kenny, Kristine Yaffe, Juan Llibre Rodriguez

**Affiliations:** ^1^Global Brain Health Institute, Trinity College Dublin, Dublin, Ireland; ^2^Global Brain Health Institute, University of California San Francisco, San Francisco, CA, United States; ^3^Department of Neurology, Washington University School of Medicine, St. Louis, MO, United States; ^4^Department of Psychiatry, Mercer's Institute for Successful Ageing, St. James's Hospital, Dublin, Ireland; ^5^The Irish Longitudinal Study on Ageing, Trinity College Dublin, Dublin, Ireland; ^6^The Irish Longitudinal Study on Ageing, Trinity College Dublin, Dublin, Ireland; ^7^Department of Psychiatry, Neurology and Epidemiology, University of California, San Francisco, San Francisco, CA, United States; ^8^Facultad de Medicina Finley–Albarrán, Universidad de Ciencias Médicas de la Habana, Havana, Cuba

**Keywords:** dementia, risk profile, lifestyle, older adults, epidemiology

## Abstract

**Introduction:** Little is known about risk factors of dementia in Latin American countries. We aimed to identify socio–demographic, health and lifestyle risk factors of incident dementia in Cuban older adults.

**Methods:** Data were from 1,846 participants in the Cuban cohort of the 10/66 Dementia Research Group. Participants completed questionnaires, health examinations, and cognitive tests at baseline (2003–2006) and 4.5 years later (2007–2010). Associations between risk factors (baseline) and incident dementia (follow-up) were examined using logistic regression.

**Results:** Just over 9% of participants developed dementia. Overall, older age and low physical activity were associated with incident dementia. In those 65–74 years of age, depression, stroke and low physical activity were associated with incident dementia. In those ≥75 years of age, low physical activity, never eating fish, and smoking were associated with incident dementia.

**Conclusions:** Modifiable lifestyle factors play an important role in developing dementia in Cuban older adults. This knowledge opens up opportunities for preventive strategies.

## Introduction

Two-thirds of the people living with dementia live in low- and middle-income countries (LMIC) (1). Due to population aging and changing lifestyles, the prevalence of dementia risk factors such as midlife hypertension and diabetes is rapidly increasing in these countries (2, 3). Over the coming decades, the largest increase in dementia prevalence will be in LMIC (1, 4). In 2015, 27.3 million people lived with dementia in LMIC. This number is projected to increase to 89.3 million in 2050 (1). Until there is a cure, implementation of evidence-based preventive strategies is crucial to reduce the impact of dementia on the society and the economy. There is strong evidence that the dementia risk in populations can be lowered by reducing the prevalence of risk factors (5, 6).

While many researchers have examined risk factors of dementia in high income countries (HIC), little is known about risk factors for dementia in LMIC. Findings from HIC cannot necessarily be extrapolated to LMIC as the prevalence of dementia and established risk factors differ between LMIC and HIC (7, 8). A study in which population attributable fractions (PAF) were estimated for nine health and lifestyle factors, showed that for seven of the nine risk factors the PAF was much higher in Latin America than worldwide (6, 9). Moreover, the overall PAF was much higher in Latin America [55.8%, confidence interval [CI]: 54.9–56.7] than worldwide (35%, CI: 34.1–35.9), suggesting that health and lifestyle factors may contribute more to the dementia risk in Latin American countries than in other countries (9). Previous Latin American studies have shown associations between individual risk factors, such as tobacco use and APOE genotype, and dementia (10, 11). In two studies in Mexico (12) and Argentina (13) risk profiles of cognitive impairment were examined, but a comprehensive analysis of factors associated with increased dementia risk is lacking.

Given the absence of a cure and limited resources for healthcare in Cuba, the policy focus is on prevention. To guide preventive strategies, it is pivotal that we know which risk factors are most important for focused health promotion programs in Cuba. The aim of the present study was to identify risk factors for incident dementia in Cuban older adults. The focus was on socio-demographic, health and lifestyle factors, with a particular interest to identify potentially modifiable factors.

## Methods

### Setting and Study Design

The data used in this study were from the Cuban cohort of the 10/66 Dementia Research Group, a population-based cohort study designed to examine the prevalence and determinants of stroke, dementia and mortality in LMIC (14, 15). For the current analyses, data were used from the first (2003–2005) and second (2007–2010) data collection waves, that involved clinical and informant interviews, physical examination, and blood draws collected by trained interviewers (15). The variables were measured using the same methods across the data collection waves. Where available, translated, culturally adapted and validated instruments were used (see §2.3 for details per variable).

Written informed consent was obtained from all participants. The design of the 10/66 study and protocols for data collection were described in detail elsewhere (14) and were approved by the Medical Ethics committee of the University of Havana and the King's College London research Ethics Committee. Additional ethics approval for the current study was obtained from the Medical Ethics committee of the University of Havana and the Trinity College Dublin Faculty of Health Sciences Ethics Committee.

### Participants

**Figure 1:**
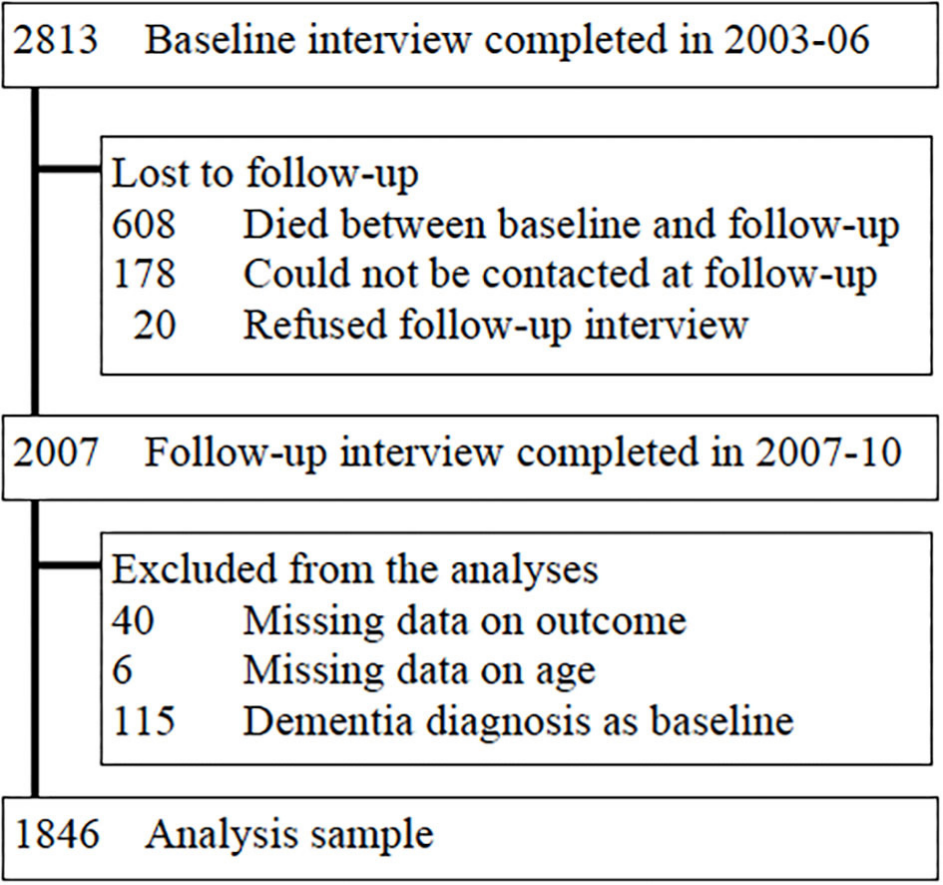
Flow chart. Describing the flow of participants in the study, attrition, and selection of participants in the analyses sample.

All residents aged 65 years and over in geographically defined catchment areas (urban and rural sites in Cuba) were invited to participate through door knocking (94% response rate) (14). Baseline surveys were completed by 2,813 participants in 2003–2005. Of these, 806 participants were lost to follow up (see Figure 1 for further details) and 2007 participants completed the follow-up interview in 2007–2010 (average follow-up of 4.5 years, range 2–7) Participants with a dementia diagnosis at baseline (*n* = 115) or missing data on dementia diagnosis (*n* = 40) or age (*n* = 6) were excluded from the analyses. Therefore, data from 1,846 participants were included in the current analyses.

### Measures

#### Incident Dementia

Incident dementia was operationalized using two methods previously developed and validated by the 10/66 Dementia Research Group (16, 17). The first method is a regression algorithm based upon: (a) a structured Geriatric Mental State interview (18), (b) cognitive tests including the Community Screening Instrument for Dementia (CSI-D) (19), verbal fluency task, object recognition and modified CERAD 10 word list learning task with delayed recall (20) and; (c) an informant interview (CSI-D) (19) for evidence of cognitive and functional decline. Participants scoring above a prediction probability cutpoint are defined as having dementia. The second method is based on operational definitions of the DSM IV criteria (21). Although the DSM-IV does not specify a criterion for the diagnosis of dementia, this could be inferred from the common elements of the DSM-IV criteria for each of the dementia sub-type diagnoses (21). The criteria for DMS-IV diagnosis were: impairment in memory and at least one other domain of cognitive function; impairment in social or occupational functioning, and representing a decrease from a previous level of functioning; not occurring exclusively during delirium; and not better accounted for by another mental disorder. Validation of the two definitions of dementia in the 10/66 cohort, demonstrated that the DSM-IV criterion was specific but insensitive to mild to moderate dementia (16). The 10/66 dementia algorithm corresponded better to clinician diagnosis and was more sensitive to milder cases (16). To capture the maximum number of cases with dementia, all participants who met the criteria for at least one of the two definitions were classified as having dementia. Participants with dementia at baseline were excluded from the analyses. Participants with new onset dementia at follow-up were classified as having incident dementia.

#### Sociodemographic Factors

The socio-demographic variables were based on self- or informant-report and included: age (≤75 years and >75 years), sex, level of education [completed primary or less, secondary, or tertiary education [i.e., college/university]], marital status (response options collapsed as married/cohabiting; widowed/divorced/separated; never married) and occupational class (professional trade; skilled laborer; laborer).

#### Health Factors

Hypertension was measured and dichotomized using the ISH criterion of ≥140 mmHg for systolic and ≥90 mmHg for diastolic bloodpressure. Waist circumference was measured using standard procedures and obesity was defined as a waist circumference of >102 cm. Total and LDL cholesterol were derived from blood samples and high levels were defined as levels exceeding the 75th percentile in the distribution (total: ≥6 mmol/L, LDL: ≥4.3 mmol/L). Presence of doctor diagnosed diabetes, stroke, head trauma (i.e., head injury with loss of conscious) and ischemic heart problems (i.e., myocardial infarction and/or angina) were based on self-report. Depression was measured using the Geriatric Mental State Examination, and its computerized algorithm AGECAT, which provided International Classification of Disease 10 (ICD10) depressive episode diagnoses (15). Hearing and eye problems were based on self-report and were considered present if these caused at least some difficulty in daily life. The presence of sleep complaints was based on the question “Have you had trouble sleeping recently?” Family history of dementia were based on self-report. Cognitive function was based on a sum score of the cognitive test battery.

#### Lifestyle Factors

Lifestyle factors were based on self-report or informant report if severely demented. Participants were asked to report the maximum usual consumptions by type of drink per week before and after the age of 65 years. Hazardous drinking was defined as 14 units/week for women and 21 units/week for men (with one unit being defined as a small glass of beer, a single measure of spirits [32 units per bottle], or one glass of wine or sherry). Smoking status was defined as never or ex-smoker vs. current smoker. Physical activity was assessed with the question: “Taking into account both work and leisure, would you say that you are: very, fairly, not very, or not at all physically active?” Participants were asked to report the frequency of fish and meat consumption (response options were: every day, some days, most days, or never) and the number of fruit and vegetable servings in the past 3 days (categorized as ≤3, 4–8, and ≥9).

### Statistical Analysis

Descriptive statistics were used to describe and compare baseline characteristics of participants (i) with and without incident dementia, and (ii) whose data were included in the analyses with those who were lost to follow-up. While 40.8% of the sample had missing data on at least one risk factor, only two variables had more than 10% missing data. Multiple imputation by chained equations was used to impute missing values and 20 datasets were created (22). Pooled results are presented for the main analyses. Logistic regression was used to examine associations between potential risk factors and incident dementia. Univariable models were run for each potential risk factor separately, with adjustment for sex and education. The multivariable models included age, sex, education and all risk factors with odds ratios (OR) of ≤ 0.75 or ≥1.4 in the univariable models. These cut-points were based on effect size rather than *p*-values or confidence intervals (CI), as we wanted to prioritize clinical relevance over statistical significance. As previous studies have shown that the strength of associations between risk factors and dementia varies with age (23), the findings are presented for the total sample and separately for the age-groups 65–74 and ≥75 years.

## Results

Data from 1,846 participants without dementia at baseline were used for the current analyses (Figure 1). Participants whose data were included were younger, healthier and had better lifestyles than participants who were lost to follow-up (Figure 1, Appendix Table A). Of the included participants, 169 (9.2%) developed dementia between baseline and follow-up. Participants who developed dementia were older (*p* < 0.001), less likely to have completed tertiary education (*p* = 0.002), and more likely to be widowed, divorced, or separated (*p* < 0.001) at baseline than those who did not develop dementia. Moreover, they had poorer cognition, less healthy lifestyles and more health problems (Table 1). Among the 1,132 participants who were 65–74 years old at baseline, 61 (5.4%) had incident dementia. Among the 714 participants who were ≥75 years old at baseline, 108 (15.1%) had incident dementia.

**Table 1:** Baseline characteristics of participants with and without incident dementia in the Cuban cohort of the 10/66 study (*n* = 1,846).

	***n***	**No Dementia (*n* = 1,677)**	**Incident dementia (*n* = 169)**	***p-value***
Age (M ± SD)	1,846	73.1 ± 5.9	77.8 ± 6.5	< 0.001
Sex (*n*, %)	1,846			0.17
Female		1,103 (65.8)	120 (71.0)	
Male		576 (34.2)	49 (29.0)	
Education (*n*, % level completed)	1,843			0.002
Tertiary/college		310 (18.5)	15 (8.9)	
Secondary		473 (28.3)	43 (25.4)	
None/primary		891 (53.2)	111 (65.7)	
Marital status (n, %)	1,843			< 0.001
Married/cohabiting		787 (47.0)	54 (32.0)	
Widowed/divorced/separated		738 (44.1)	102 (60.4)	
Never married		149 (8.9)	13 (7.7)	
Occupational class (*n*, %)	1,753			0.02
Professional (1–3)		675 (42.3)	48 (30.8)	
Trade (4,5)		206 (12.9)	28 (18.0)	
Skilled laborer (6,7)		452 (28.3)	55 (35.3)	
Laborer (8,9)		264 (16.5)	25 (16.0)	
Hypertension (*n*, %)	1,842	960 (57.4)	109 (64.5)	0.07
Obesity (*n*, %)	1,838	694 (41.6)	53 (31.6)	0.01
High total cholesterol (*n*, %)	1,482	374 (28.0)	40 (27.0)	0.80
High LDL cholesterol (*n*, %)	1,126	239 (23.6)	33 (28.7)	0.23
Diabetes (*n*, %)	1,840	299 (17.9)	28 (16.6)	0.67
Depression (*n*, %)	1,827	458 (27.6)	61 (36.8)	0.01
Stroke (*n*, %)	1,842	80 (4.8)	17 (10.1)	0.003
Ischemic heart problem (*n*, %)	1,844	225 (13.4)	18 (10.7)	0.31
Head Trauma (*n*, %)	1,839	91 (5.4)	8 (4.8)	0.72
Hearing problem (*n*, %)	1,843	132 (7.9)	22 (13.0)	0.02
Eye problem (*n*, %)	1,840	436 (26.1)	62 (36.7)	0.003
Current high-risk alcohol use (*n*, %)	1,822	48 (2.9)	7 (4.1)	0.37
Past high-risk alcohol use (*n*, %)	1,824	107 (6.5)	13 (7.7)	0.54
Smoking (*n*, % current smoker)	1,842	313 (18.7)	30 (17.8)	0.76
Physical activity (*n*, %)	1,840			< 0.001
Highly active		515 (30.8)	27 (16.1)	
Somewhat active		777 (46.5)	81 (48.2)	
Not (very) active		380 (22.7)	60 (35.7)	
Fish consumption (*n*, % never)	1,842	129 (7.7)	23 (13.6)	0.008
Meat consumption (*n*, % never/some days)	1,842	1,077 (64.4)	112 (66.3)	0.62
Fruit & vegetable servings	1,843			0.001
9 or more in last 3 days		322 (19.2)	19 (11.2)	
4–8 in last 3 days		683 (40.8)	58 (34.3)	
3 or fewer in last 3 days		669 (40.0)	92 (54.4)	
Sleep complaints (n, %)	1,842	567 (33.9)	50 (29.6)	0.26
Family history (*n*, %)	1,842	291 (17.4)	38 (22.5)	0.10
Cognitive function (Md [IQR])	1,846	31.2 [30.0–32.1]	29.8 [27.7–31.2]	< 0.001

*IQR, interquartile range; M, mean; Md, median; n, numer of participants with available data; SD, standard deviation*.

*Normally distributed continuous variables were described as means and standard deviation and groups were compared using the t-test. Not-normally distributed continuous variables were described as median and interquartile range and groups were compared using the Wilcoxon singed rank test. Categorical variables were described as numbers and percentages, and groups were compared using the Chi-squared test*.

In the total sample, statistically significant univariable associations with incident dementia were found for the risk factors age, education, marital status, obesity, stroke, depression, physical activity, fish consumption, fruit and vegetable consumption and family history (Table 2). In the multivariable model, these associations remained statistically significant for the risk factors age (≥75 years vs. 65–74 years: OR = 2.70, 95% CI = 1.90–3.84), marital status (widowed/divorced/separated vs. married/cohabiting: OR = 1.63, 95% CI = 1.10–2.41), physical activity (somewhat active vs. highly active: OR = 1.81, 95% CI = 1.13–2.90; not (very) active vs. highly active: OR = 2.29, 95% CI = 1.49–4.16), fish consumption (regular vs. rarely: OR = 1.77, 95% CI = 1.06–2.95) and fruit and vegetable consumption (≤ 3 vs. ≥9 servings: OR = 1.96, 95%CI = 1.15–3.35) (Table 3).

**Table 2:** Univariable associations between potential risk factors and incident dementia in the total sample (*n* = 1,846), and stratified by age.

	**Total sample**		**65–74 years**		**≥75 years**	
	**OR**	95% CI^*^	**OR**	95% CI^*^	**OR**	95% CI^*^
**Age**						
65–74 years	1					
≥75 years	2.87	2.05–4.02				
**Sex**						
Women	1		1		1	
Men	0.85	0.59–1.21	0.88	0.50–1.54	0.83	0.53–1.32
**Education**						
Tertiary	1		1		1	
Secondary	1.62	0.88–2.99	2.10	0.87–5.07	1.18	0.50–2.78
None/primary	1.85	1.05–3.27	2.57	1.12–5.88	1.30	0.59–2.83
**Marital status**						
Married/cohabiting	1		1		1	
Widowed/divorced/separated	1.70	1.16–2.48	1.79	1.00–3.19	1.63	0.99–2.69
Never married	1.13	0.60–2.15	1.62	0.64–4.10	0.84	0.35–2.03
**Occupational class**						
Professional (1–3)	1		1		1	
Trade (4,5)	1.48	0.88–2.50	1.43	0.63–3.23	1.55	0.77–3.11
Skilled laborer (6,7)	1.26	0.79–2.00	1.41	0.69–2.88	1.19	0.65–2.18
Laborer (8,9)	0.98	0.56–1.69	0.85	0.34–2.15	1.06	0.53–2.12
Hypertension	1.25	0.89–1.74	1.30	0.77–2.22	1.21	0.79–1.86
Obesity	0.62	0.44–0.89	0.56	0.32–0.99	0.67	0.42–1.05
Total cholesterol	0.98	0.67–1.43	1.23	0.69–2.18	0.83	0.50–1.37
LDL cholesterol	1.31	0.86–1.97	1.64	0.91–2.95	1.09	0.62–1.92
Diabetes	0.91	0.59–1.40	1.24	0.67–2.32	0.71	0.38–1.29
Head Trauma	0.96	0.45–2.04	0.87	0.26–2.86	1.03	0.39–2.74
Stroke	2.16	1.23–3.79	3.38	1.51–7.61	1.55	0.72–3.35
Ischemic heart problems	0.70	0.42–1.17	0.22	0.05–0.90	1.00	0.56–1.79
Depression	1.49	1.06–2.09	1.90	1.11–3.27	1.27	0.81–1.98
Hearing problem	1.32	0.80–2.16	0.98	0.30–3.26	1.41	0.81–2.44
Eye problem	1.39	0.99–1.95	1.02	0.56–1.86	1.64	1.08–2.50
Current high–risk alcohol use	2.24	0.96–5.26	2.53	0.91–7.00	1.71	0.34–8.54
Past high–risk alcohol use	1.60	0.83–3.07	2.17	0.92–5.10	1.11	0.40–3.07
Smoking status	1.29	0.83–2.01	0.77	0.40–1.50	2.17	1.19–3.96
**Physical activity**						
Highly active	1		1			1
Somewhat active	1.76	1.11–2.77	2.80	1.33–5.92	1.26	0.70–2.26
Not (very) active	2.47	1.52–4.01	2.77	1.20–6.42	2.17	1.19–3.95
Never eating fish	1.77	1.09–2.87	1.01	0.39–2.59	2.30	1.27–4.15
Never/some days eating meat	0.94	0.67–1.32	0.84	0.48–1.46	1.01	0.66–1.56
**Fruit & vegetable servings**						
9 or more in last 3 days	1		1		1	
4–8 in last 3 days	1.34	0.78–2.30	1.80	0.72–4.54	1.10	0.56–2.17
3 or fewer in last 3 days	2.16	1.28–3.62	2.88	1.19–7.00	1.77	0.92–3.39
Sleep complaints	0.74	0.52–1.05	0.70	0.39–1.26	0.75	0.48–1.18
Family history	1.53	1.03–2.26	1.67	0.92–3.03	1.41	0.83–2.37

*OR odd ratio; 95%CI 95% confidence interval*.

* *Associations were adjusted for sex and education*.

**Table 3:** Multivariable models for the total sample (*n* = 1,846), and stratified by age.

	**Total sample**		**65–74 years**		**≥75 years**	
	**OR**	**95% CI**	**OR**	**95% CI**	**OR**	**95% CI**
**Age**						
65–74 years	1					
≥75 years	2.70	1.90–3.84				
**Sex**						
Women	1		1		1	
Men	0.84	0.54–1.29	0.78	0.39–1.56	0.88	0.50–1.56
**Education**						
Tertiary	1		1		1	
Secondary	1.72	0.90–3.31	2.29	0.89–5.87	1.16	0.45–2.99
None/primary	1.74	0.90–3.38	2.44	0.91–6.55	1.15	0.45–2.93
**Marital status**						
Married/cohabiting	1		1		1	
Widowed/divorced/separated	1.63	1.10–2.41	1.81	0.98–3.35	1.56	0.92–2.65
Never married	0.87	0.45–1.70	1.51	0.56–4.05	0.61	0.24–1.54
**Occupational class**						
Professional (1–3)	1		1		1	
Trade (4,5)	1.47	0.86–2.53	1.33	0.56–3.13	1.59	0.76–3.30
Skilled laborer (6,7)	1.14	0.70–1.85	1.15	0.53–2.48	1.11	0.58–2.11
Laborer (8,9)	0.77	0.43–1.40	0.67	0.25–1.79	0.82	0.38–1.75
**Hypertension**						
Obesity	0.63	0.43–0.91	0.58	0.32–1.05	0.64	0.39–1.03
**Total cholesterol**						
LDL cholesterol			1.66	0.89–3.08		
Diabetes						
**Head trauma**						
Stroke	1.81	1.00–3.28	3.08	1.25–7.58	1.41	0.62–3.22
Ischemic heart problems	0.71	0.42–1.21	0.23	0.05–0.99		
Depression	1.39	0.96–2.00	1.80	1.02–3.19		
Hearing problem					1.04	0.56–1.91
Eye problem					1.48	0.94–2.32
Current high–risk alcohol use	2.10	0.68–6.49	1.52	0.34–6.67	1.44	0.26–7.84
Past high–risk alcohol use	1.27	0.54–2.99	1.86	0.56–6.17		
Smoking status					2.21	1.17–4.16
**Physical activity**						
Highly active	1		1		1	
Somewhat active	1.81	1.13–2.90	2.74	1.27–4.36	1.24	0.67–2.28
Not (very) active	2.29	1.49–4.16	2.67	1.08–6.59	2.05	1.06–3.94
Never eating fish	1.77	1.06–2.95			2.44	1.28–4.66
**Fruit & vegetable servings**						
9 or more in last 3 days	1		1		1	
4–8 in last 3 days	1.26	0.72–2.21	1.68	0.65–4.36	1.07	0.53–2.16
3 or fewer in last 3 days	1.96	1.15–3.35	2.22	0.88–5.59	1.81	0.92–3.57
Sleep complaints	0.62	0.42–0.90	0.57	0.30–1.06		
Family history	1.47	0.98–2.22	1.70	0.91–3.19	1.42	0.82–2.48

*OR Odds ratio, CI 95% confidence interval*.

*Multivariable models include age, sex, education, plus all risk factors with ORs <0.75 or >1.40 in the univariable models (Table 2)*.

In the younger age-group (65–74 years), statistically significant univariable associations with incident dementia were found for the risk factors education, marital status, obesity, stroke, ischemic heart problems, depression, physical activity, and fruit and vegetable consumption (Table 2). In the multivariable model, these associations remained statistically significant for the risk factors stroke (yes vs. no: OR = 3.08, 95% CI = 1.25–7.58), ischemic heart problems (yes vs. no: OR = 0.23, 95% CI = 0.05–0.99), depression (yes vs. no: OR = 1.80, 95% CI = 1.02–3.19) and physical activity (somewhat active vs. highly active: OR = 2.74, 95% CI = 1.27–4.36; not (very) active vs. highly active: OR = 2.67, 95% CI = 1.08–6.59) (Table 3).

In the older age-group (≥75 years), statistically significant univariable associations with incident dementia were found for the risk factors eye problems, smoking status, physical activity and fish consumption (Table 2). In the multivariable model, these associations remained statistically significant for the risk factors smoking status (current vs. never/ex-smoker: OR = 2.21, 95% CI = 1.17–4.16), physical activity (not (very) active vs. highly active: OR = 2.05, 95% CI = 1.06–3.94) and fish consumption (regular vs. rarely: OR = 2.44, 95% CI = 1.28–4.66) (Table 3).

## Discussion

This study is the first to present a comprehensive identification of factors associated with incident dementia in Cuban older adults. The results show that the risk profiles are different for adults aged 65–74 years and those aged ≥75 years. The risk factors for which statistically significant associations were found with incident dementia, were predominantly health factors in the younger age group (i.e., stroke, ischemic heart problems, depression) and lifestyle factors in the older age group (i.e., smoking, physical activity, and fish consumption).

### Changes in Risk Profiles With Age

The difference in risk profiles between the younger and the older age groups is in line with previous studies in which changes were found in associations between risk factors and dementia with age. For some risk factors, associations seem to become stronger with age (e.g., smoking) (24), whereas for other risk factors associations seem to weaken with age (e.g., APOεE4, blood pressure) (25–28). Several explanations may be possible. First, lifestyle has a delayed, long term effect on health in general and brain health in particular. Accumulation of lifestyle across the lifespan, particularly during midlife, may be more important than the lifestyle at a given point in time. Second, at older ages, the higher prevalence of other chronic conditions may weaken associations between risk factors and dementia (23), particularly for risk factors that are common across chronic conditions, such as lifestyle factors. Third, mixed patterns of dementia-pathology are more common at older ages (29). Different risk factors may be relevant for different dementia-pathologies and more difficult to identify in mixed pathologies. This may also explain why fewer risk factors were found in the older age group than in the younger age group.

### Lifestyle Factors

In line with previous studies that attributed a large part of the dementia risk to lifestyle factors (9), lifestyle factors, particularly physical activity, dominate the risk profiles in this cohort of Cuban older adults. Previous studies using data from the wider Latin American 10/66 cohort including data from Dominican Republic, Mexico, Peru, Puerto Rico, Venezuela and Cuba (11), have demonstrated that better scores on a cardiovascular health index (based on physical activity, smoking, alcohol, hypertension, obesity, cholesterol, glucose, and intake of meat, fish, fruits and vegetables) was associated with lower risk of dementia (30). The current study adds that, when each factor is viewed independently, some but not all of these factors are associated with dementia and the associations differ for the younger and older age groups. These collective findings suggest that either the association of the cardiovascular health index with dementia risk is driven by individual factors rather than the overall cardiovascular burden, or that some individual factors are important only in combination with other factors.

The finding that never eating fish increases the risk of dementia in the older age group is consistent with findings from previous cross-sectional studies in Latin America (31) and other LMIC (32). The beneficial effects of fish consumption are attributed to the salutary effects of long-chain omega-3 polyunsaturated fatty acids on neurone membranes, vascular anti-inflammatory properties and neuroplasticity (33).

### Health Factors

Stronger associations with dementia were found with stroke and depression in the younger age group, and with eye problems in the older age group. As explained above, the attenuation of the associations between chronic conditions and dementia with age may be explained by the higher prevalence of chronic conditions at older ages (23) and therefore reduced contrast between groups with and without dementia. Also, chronic conditions acquired at younger ages may reflect longer exposure to common underlying risk factors.

The association between depression and incident dementia has been previously demonstrated in the wider Latin American 10/66 cohort (34). That study identified substantial variation between the countries in strength of associations, with the strongest associations found in Cuba (Hazard Ratio [HR]: 2.48, CI: 1.52–4.06) and Venezuela (HR: 2.12, CI: 1.16–3.87) and no associations found in the Dominican Republic (HR: 1.01, CI: 0.62–1.62) and Puerto Rico (HR: 0.81, CI: 0.19–3.48). The researchers cited differences in prevalence of depression as the main explanation for these variations (34). It may be interesting to explore if these variations between countries remain after stratification by age group.

Consistent with other studies, both in LMIC (35) and HIC (36), stroke was a strong predictor of dementia, particularly in the younger age group. Similar to previous Latin American studies, diabetes was not associated with dementia (37). Contrary to our expectations, ischemic heart disease was associated with a reduced risk of dementia in the younger age group. This likely results from survival bias as participants with ischemic heart disease have higher mortality rates than participants without ischemic heart disease and the risk of dementia increases with age.

Hearing loss and vision loss have been associated with dementia in HIC (38–42), but evidence for these associations in Latin America or other LMIC is lacking. The current results suggest that eye problems, but not hearing problems, are associated with an increased risk of dementia in Cuban older adults, but only in the older age group. Two theories that may explain the associations between hearing and vision problems and dementia are the causal pathway and the shared etiological pathway (39). Panza et al. state there is currently no epidemiological evidence to support a causal pathway between hearing loss and dementia, but argue that more research is needed to test these theories (39). Some evidence for a shared etiological pathway between eye problems and dementia comes from a review in which the researchers described the common underlying cardiovascular risk factors for cataract and dementia (43).

In a recent systematic review, the researchers found that insomnia was associated with a higher risk of Alzheimer's disease (three studies, pooled OR = 1.51, CI = 1.06–2.41) (44), but not with all-cause dementia (12 studies, pooled OR = 1.17, CI = 0.95–1.43) or vascular dementia (four studies, pooled OR = 1.13, IC = 0.94–1.35). In contrast, we found that sleep complaints were associated with a lower risk of dementia in the total sample (OR = 0.63, CI = 0.43–0.92). It may be that the question we used was insufficiently sensitive to pick up sleep disturbances. Also, participants who were already experiencing some cognitive decline at baseline (but did not yet meet criteria for dementia) may have been less likely to report sleeping complaints than participants with good cognitive function, resulting in reporting bias. The findings may also partly be explained by residual confounding.

### Comparison With Risk Profiles in Other Latin American Countries

Few researchers to date have examined risk factors for dementia or cognitive impairment in Latin American countries. In a prospective study among 3002 Mexican older adults (aged 60+), risk factors were identified for severe cognitive impairment (defined as low score on the Cross-Cultural Cognitive Examination and difficulties with daily activities) (12). A cross-sectional study among 1453 Argentinean older adults (aged 60+) examined correlations between risk factors and cognitive impairment (defined as MMSE ≤ 22) (13). Comparison of the risk profiles identified in these two studies and the current study reveals some overlap but also some differences. In all three cohorts, higher age and low education were associated with dementia or cognitive impairment. Inactivity was associated with dementia or cognitive impairment in the Argentinean and Cuban cohorts, but not in the Mexican cohort. Stroke was associated with dementia or cognitive impairment in the Mexican and Cuban cohorts, but not in the Argentinean cohort. Diabetes was associated with cognitive impairment in the Mexican cohort only. Head trauma was associated with cognitive impairment only in the Argentinean cohort. Depression was associated with dementia only in the Cuban cohort. In the current study, we additionally identified not eating fish, fruit and vegetables as a risk factor for dementia. Differences in study design, sample characteristics and definition of the outcome likely explain variations in the findings across the three cohorts. The differences in risk profiles also suggest that intervention strategies require tailoring to the characteristics of the country.

### Implications for Health Promotion

The current findings confirm that lifestyle and health factors are important contributors to the risk of dementia in Cuban older adults. The finding that lifestyle factors are important at older ages highlight that lifestyle interventions should not only focus on midlife, but should be continued in older ages. In the younger age group, depression and stroke were important risk factors for dementia. Given the large overlap in risk factors for stroke and dementia, health promotion programs targeting stroke will also help delay the onset of dementia and vice versa. Whether depression causes dementia, is a consequence of dementia or the two conditions are simply coinciding, is still up for debate (45, 46). We recommend that future studies evaluating depression treatment should include cognitive outcomes to examine whether adequate treatment of depression can lower dementia risk.

### Strengths and Limitations

While the 10/66 cohort offers the best available data on incident dementia and its risk factors in Cuba, the study was relatively underpowered. Interpretation of the findings should take into account the relatively small sample and number of participants with incident dementia during the 4.5 years follow-up, resulting in wide confidence intervals. To maintain the maximum amount of available information and representativeness of the sample, we imputed missing values. Most missing values were due to blood samples being taken only in a subsample. About a third of participants (*n* = 806) were lost to follow-up; the main reason being death (*n* = 608). Participants whose data were included in the analyses were younger, healthier, and had better lifestyles. Hence the current results are representative for a somewhat more vital community-dwelling older population. While this study benefited from the comprehensive assessment of potential risk factors in this cohort, we may have missed factors that were not measured, for example, cognitive stimulation, social activity, other dietary components, exposure to air pollution and traumatic life events (47–49).

## Conclusion

In conclusion, risk profiles for incident dementia differ for 65–74 year old adults and ≥75 year old adults in Cuba. In the younger age group, education, depression, stroke, and physical activity were associated with a higher dementia risk. In the older age group, smoking, physical activity, and not eating fish were associated with a higher dementia risk. Thus, modifiable lifestyle factors play an important role in developing dementia in Cuban older adults, even at higher ages. This knowledge opens up opportunities for development and implementation of preventive strategies. Preventive strategies may require tailoring to age groups.

## Data Availability Statement

Publicly available datasets were analyzed in this study. A request for access to the 10/66 Dementia Research Group data can be submitted to dementiaresearchgroup1066@kcl.ac.uk.

## Author Contributions

GP, JLG, KY, and JLR were responsible for the study design. GP and AA were responsible for the data analyses. GP was responsible for drafting the manuscript. All authors contributed to interpretation of the findings, provided critical feedback on drafts of the manuscript, and approved the final manuscript.

## Conflict of Interest

The authors declare that the research was conducted in the absence of any commercial or financial relationships that could be construed as a potential conflict of interest.
