# Supplementary material for: Risk Factors for Incident Dementia Among Older Cubans
Source: Front Public Health. 2020 Sep 10;8:481. doi: 10.3389/fpubh.2020.00481 (PMC7511701; doi:10.3389/fpubh.2020.00481)
Supplement: Supplementary file 1 [file Data_Sheet_1.docx]

Appendix Table A. Baseline characteristics of participants whose data were included in the current analyses compared with participants were lost to follow-up.

|  | Included  (n=1846) | Lost to follow-up  (n=805) | p-value |
| --- | --- | --- | --- |
| Age (M±SD) | 73.5 (6.1) | 78.4 (7.5) | <0.001 |
| Sex (n, % female) | 66.3 | 62.5 | 0.06 |
| Education (n, % level completed) |  |  | <0.001 |
| Tertiary/college | 17.6 | 15.4 |  |
| Secondary | 28.0 | 20.6 |  |
| None/primary | 54.4 | 64.1 |  |
| Marital status (n, %) |  |  | <0.001 |
| Married/cohabiting | 45.6 | 36.9 |  |
| Widowed/divorced/separated | 45.6 | 52.9 |  |
| Never married | 8.8 | 10.2 |  |
| Occupational class (n, %) |  |  | 0.002 |
| Professional (1-3) | 41.2 | 35.1 |  |
| Trade (4-5) | 13.4 | 16.0 |  |
| Skilled laborer (6-7) | 28,9 | 27.4 |  |
| Laborer (8-9) | 16.5 | 21.5 |  |
| Hypertension (n, %) | 58.0 | 58.5 | 0.81 |
| Obesity (n, %) | 40.6 | 30.6 | <0.001 |
| High total cholesterol (n, %) | 5.4 (1.2) | 5.2 (1.2) | 0.03 |
| High LDL cholesterol (n, %) | 3.5 (1.2) | 3.4 (1.2) | 0.05 |
| Diabetes (n, %) | 17.8 | 20.7 | 0.08 |
| Depression (n, %) | 28.4 | 31.2 | 0.15 |
| Stroke (n, %) | 5.3 | 12.8 | <0.001 |
| Ischemic heart problem (n, %) | 13.2 | 14.6 | 0.34 |
| Head Trauma (n,%) | 5.4 | 5.9 | 0.62 |
| Hearing problem (n, %) | 8.4 | 13.0 | <0.001 |
| Eye problem (n, %) | 27.1 | 37.0 | <0.001 |
| Current high-risk alcohol use (n, %) | 3.0 | 3.3 | 0.70 |
| Past high-risk alcohol use (n, %) | 6.6 | 8.0 | 0.20 |
| Smoking (n, % current smoker) | 18.6 | 21.5 | 0.08 |
| Physical activity (n, %) |  |  | <0.001 |
| Highly active | 29.5 | 16.0 |  |
| Somewhat active | 46.6 | 35.6 |  |
| Not (very) active | 23.9 | 48.4 |  |
| Fish consumption (n, % never) | 8.3 | 12.9 | <0.001 |
| Meat consumption (n, % never/some days) | 35.5 | 40.2 | 0.02 |
| Fruit & vegetable servings |  |  | 0.002 |
| 9 or more in last 3 days | 18.5 | 13.9 |  |
| 4-8 in last 3 days | 40.2 | 38.5 |  |
| 3 or fewer in last 3 days | 41.3 | 47.7 |  |
| Sleep complaints (n, %) | 33.5 | 34.0 | 0.80 |
| Family history (n, %) | 17.9 | 16.7 | 0.48 |
| Cognitive function (Md [IQR]) | 31 [30-32] | 30 [26-31] | <0.001 |

IQR interquartile range; M mean; Md median; n number of participants with available data; SD standard deviation

Normally distributed continuous variables were described as means and standard deviation and groups were compared using the t-test. Not-normally distributed continuous variables were described as median and interquartile range and groups were compared using the Wilcoxon singed rank test. Categorical variables were described as numbers and percentages, and groups were compared using the Chi-squared test.
